# Supplementary material for: Identification of photoperiod-induced specific miRNAs in the adrenal glands of Sunite sheep (Ovis aries)
Source: Front Vet Sci. 2022 Jul 22;9:888207. doi: 10.3389/fvets.2022.888207 (PMC9354845; doi:10.3389/fvets.2022.888207)
Supplement: Supplementary file 1 [file Data_Sheet_1.ZIP › Supplementary file/miRDeep2 file.zip!/miRDeep2 file/Novel_Structure/SPLP42A3/Novel_28.pdf]

Provisional ID	:	NC_019471.2_25549
Score total	:	2.4
Score for star read(s)	:	-1.3
Score for read counts	:	0
Score for mfe	:	2.1
Score for randfold	:	1.6
Score for cons. seed	:	
Total read count	:	57
Mature read count	:	55
Loop read count	:	0
Star read count	:	2

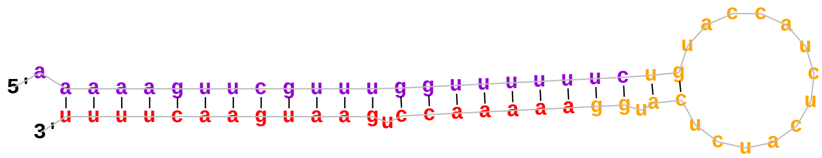

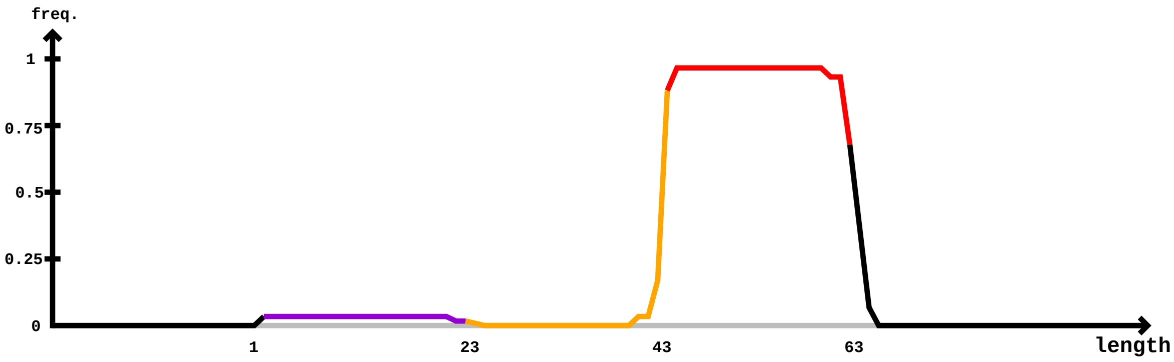

Star Mature

```
5'- acuucuguauuggguuggcuaaaaaguucguuugguuuuuucuguaccaucucaucucauggaaaaaccugaaugaacuuuuggccaacccagcagaaauuaucaaggagga  -3'  obs
    acuucuguauuggguuggcuaaaaaguucguuugguuuuuucuguaccaucucaucucauggaaaaaccugaaugaacuuuuggccaacccagcagaaauuaucaaggagga       exp
    ..((((((..((((((((((((((.(((((((((((((((((((............)).))))))))).))))))))))))))))))))))))))))...............  reads  mm  sample
    ....................aaaaaguucguuugguuuuu........................................................................  1  0  seq
    ....................aaaaaguucguuugguuuuuuc......................................................................  1  0  seq
    ...........................................................uggaaaaaccCgaaugaacu.................................  2  1  seq
    .............................................................gaaaaaccCgaaugaacuuu...............................  1  1  seq
    .............................................................gaaaaaccCgaaugaacuuuu..............................  1  1  seq
    .............................................................gaaaaaccugaaugaacuuuu..............................  4  0  seq
    .............................................................gaaaaaccAgaaugaacuuuu..............................  1  1  seq
    .............................................................gaaaaaccCgaaugaacuuuug.............................  1  1  seq
    ..............................................................aaaaaccugaaugaacCuu...............................  1  1  seq
    ..............................................................aaaaaUcugaaugaacuuu...............................  12  1  seq
    ..............................................................aaaaaccCgaaugaacuuu...............................  1  1  seq
    ..............................................................aaaaaUcugaaugaacuuuu..............................  26  1  seq
    ..............................................................aaaaaccugaaGgaacuuuu..............................  1  1  seq
    ..............................................................aaaaaccugaaugaacuuuugg............................  1  0  seq
    ...............................................................aaaaccAgaaugaacuuuu..............................  1  1  seq
    ...............................................................aaaaccugaaugGacuuuu..............................  1  1  seq
    ...............................................................aaaaUcugaaugaacuuuugg............................  2  1  seq
    ...............................................................aaaaccugaaugaacuuuugg............................  1  0  seq
```
